# Oligonucleotide-Based Therapy for FTD/ALS Caused by the *C9orf72* Repeat Expansion: A Perspective

**DOI:** 10.1155/2013/208245

**Published:** 2013-11-17

**Authors:** Stephanie A. Fernandes, Andrew G. L. Douglas, Miguel A. Varela, Matthew J. A. Wood, Yoshitsugu Aoki

**Affiliations:** ^1^Department of Physiology, Anatomy and Genetics, University of Oxford, South Parks Road, Oxford OX1 3QX, UK; ^2^Institute of Biosciences, University of Sao Paulo, Rua do Matao, 05508-090 Sao Paulo, SP, Brazil

## Abstract

Amyotrophic lateral sclerosis (ALS) is a progressive and lethal disease of motor neuron degeneration, leading to paralysis of voluntary muscles and death by respiratory failure within five years of onset. Frontotemporal dementia (FTD) is characterised by degeneration of frontal and temporal lobes, leading to changes in personality, behaviour, and language, culminating in death within 5–10 years. Both of these diseases form a clinical, pathological, and genetic continuum of diseases, and this link has become clearer recently with the discovery of a hexanucleotide repeat expansion in the *C9orf72* gene that causes the FTD/ALS spectrum, that is, c9FTD/ALS. Two basic mechanisms have been proposed as being potentially responsible for c9FTD/ALS: loss-of-function of the protein encoded by this gene (associated with aberrant DNA methylation) and gain of function through the formation of RNA *foci* or protein aggregates. These diseases currently lack any cure or effective treatment. Antisense oligonucleotides (ASOs) are modified nucleic acids that are able to silence targeted mRNAs or perform splice modulation, and the fact that they have proved efficient in repeat expansion diseases including myotonic dystrophy type 1 makes them ideal candidates for c9FTD/ALS therapy. Here, we discuss potential mechanisms and challenges for developing oligonucleotide-based therapy for c9FTD/ALS.

## 1. Introduction

Amyotrophic lateral sclerosis (ALS) is a progressive and lethal disease characterised by degeneration of motor neurons, leading to paralysis of voluntary muscles [1], culminating in respiratory failure and death within five years of disease onset [2, 3]. Frontotemporal dementia (FTD) is a cause of presenile dementia, being the second most common form of dementia in individuals younger than 65 years [4]. It is characterised by degeneration of the frontal and temporal lobes of the brain leading to changes in personality, behavior, and language, though with some preservation of perception and memory [2, 5]. FTD patients die 5–10 years after disease onset [6]. Both diseases are incurable. Recently, a hexanucleotide repeat expansion in the *C9orf72* gene has been described that is responsible for what is called the c9FTD/ALS continuum. This repeat expansion is now known to be the most common cause for familial c9FTD/ALS, and it has also been observed in apparently sporadic cases [7, 8]. The mechanism by which the repeat expansion causes disease remains to be clarified and both loss- and gain-of-function mechanisms have been proposed. Using knowledge obtained through research into antisense oligonucleotides (ASOs) therapies for diseases such as Duchenne muscular dystrophy (DMD) and myotonic dystrophy type 1, we propose that using such therapy may be a promising approach for treating *C9orf72 *repeat expansion in c9FTD/ALS.

## 2. Discovery of the *C9orf72* Mutation That Causes FTD/ALS

ALS and FTD share clinical, pathological, and genetic characteristics, supporting the hypothesis that these two illnesses form a spectrum of disease. Evidence for the existence of this spectrum comes from many different studies. For example, it is now known that up to 50% of ALS patients have some impairment of frontotemporal function and ~15% of ALS patients can specifically be said to have FTD [9–13]. In addition, significant corticospinal and lower motor neuron dysfunction have been observed across most FTD subtypes, with 10–15% of FTD patients having coexisting motor neuron disease (MND) [11, 14]. Another highly important pathological link is the recognition that transactive response DNA binding protein 43 kDa (TDP-43) aggregates can be a common pathological hallmark in both ALS and FTD [15]. 

Prior to the discovery of the *C9orf72* expansion, several genes were already known to be responsible for the development of ALS, such as superoxide dismutase 1 (*SOD1*) [16]. However, among families where both ALS and FTD cases are clustered together, strong linkage to chromosomal locus 9p21 was identified and defined as causative for the FTD/ALS spectrum [17, 18]. The associated risk haplotype has since been shown to be the same in all ALS and FTD populations studied, and is also present in affected members of several FTD/ALS families, suggesting a single founder mutation effect [19]. 

## 3. The Repeat Expansion in the *C9orf72* Gene Is Responsible for c9FTD/ALS

A study of an autosomal dominant kindred of FTD/ALS found a hexanucleotide GGGGCC repeat expansion within the *C9orf72* gene in affected individuals [7]. In their FTD patient series, the authors found that 3% of sporadic and 11.7% of familial patients carried the repeat expansion. In their ALS subjects, 4.1% of sporadic and 23.5% of familial cases had the expansion. When compared with other mutations responsible for ALS, this expansion mutation was the most common genetic cause of sporadic and familial ALS in their clinical series. In FTD, the expansion was also found to be the main cause of familial cases and of equal frequency to the progranulin (*GRN*) mutations in sporadic FTD. Unrelated carriers of the expanded repeat all shared at least one copy of the identified haplotype. The repeat length in healthy individuals ranged from 2 to 23 units, whereas the estimated size in FTD/ALS patients was 700–1600 units [7]. The minimal expansion size for the disease to occur remains undetermined, and it is important to note that genetic anticipation, which means that the phenotype is more severe at an earlier age as the disease is passed through generations and the repeat expansion increases in size, was not apparent in the majority of families studied [7]. In parallel to this study, the same repeat expansion in the *C9orf72* gene was recognized as being responsible for 46% of familial ALS and 21.1% of sporadic ALS in the Finnish population [8]. It was also found to be present in one third of familial ALS cases of outbred European origin. In addition, some 30% of the Finnish FTD cohort were found to have the mutation, making it the most common genetic cause of FTD/ALS. This study again found no evidence of anticipation; however, the data about the at-risk haplotype was slightly discordant with the study of DeJesus-Hernandez et al., [7] since they found the expansion also to be present in ALS cases without this haplotype group [8]. Following on from the publication of these original two reports, many further studies have since confirmed the presence of the *C9orf72* expansion in familial FTD/ALS cases in various different populations from around the world; for example, Gijselinck et al. also found the same repeat expansion in a Flanders-Belgian cohort study [20].

## 4. Phenotype and Pathology of c9FTD/ALS

The clinical phenotype for repeat expansion carriers includes older age of onset, shorter disease duration, psychotic symptoms, bulbar onset of ALS, and a predominantly behavioural variant of FTD [21–25]. The pathology of *C9orf72*-related disease includes TDP-43 positive inclusions and also some inclusions that are TDP-43 negative but p62/ubiquitin positive in specific parts of the brain, such as cerebellum and hippocampus [22, 25–28]. TDP-43 is a protein that is able to bind RNA, DNA, and other proteins and is mainly involved in transcription and splicing regulation [29]. Such inclusions are also found in some patients with different genetic mutations; however, classic mutations like those in *SOD1* and fused in sarcoma (*FUS*), which itself has some similarity in function to TDP-43 such as involvement in DNA repair and in regulation of transcription and RNA splicing [30], are not associated with TDP-43 pathology. The differences between *C9orf72*-associated disease and the other main genes involved in FTD and/or ALS are summarised in Table 1. 

With the discovery of *C9orf72*-related hexanucleotide expansions, much (though not all) of the previously unexplained heritability of familial ALS has been clarified. Prior to this, mutations in the *SOD1* gene were the most commonly identified cause; however, these explained at best some 20% of familial ALS cases, with mutations in other genes accounting for considerably less. In contrast, *C9orf72* expansions have now overtaken *SOD1* as the single leading identifiable cause of familial ALS [6, 31].

## 5. Potential Mechanisms of c9FTD/ALS

**Figure 1:**
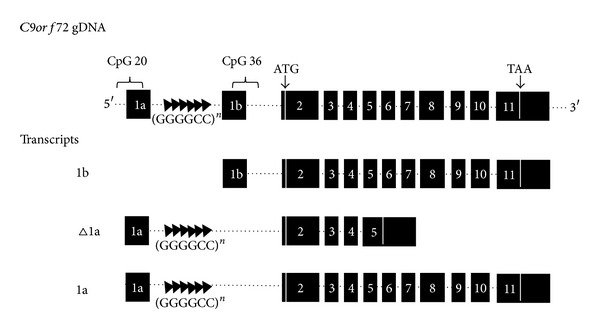
Structure of the *C9orf72* locus and its three primary transcripts. Two alternative first exons are used, 1a and 1b, and both of these lie upstream of the translation start site. Between exons 1a and 1b lies the hexanucleotide expansion region. Note that two putative CpG islands lie either side of the expansion. The shorter of these regions, CpG 20, overlaps with much of the sequence of exon 1a. Although there are three transcripts, the position of the ATG start codon in exon 2 means that only two protein isoforms are translated: isoform A is 481 amino acids long, while the shorter isoform B is only 222 amino acids in length.

The *C9orf72* gene produces three transcripts initiated from two alternative transcription start sites. Two of the transcripts comprise 11 exons but use alternative first exons, 1a or 1b. The other transcript is truncated with just 5 exons and uses a truncated version of exon 1a. Unfortunately, the naming of these transcripts has been somewhat inconsistent within the literature and among internet databases, leading to potential confusion. In this review, we therefore choose to use a clearer naming system, whereby the two longer transcripts with 11 exons are called variant 1a and variant 1b (NM_001256054 and NM_018325, resp.), while the shorter transcript is variant Δ1a (NM_145005). These three transcripts lead to the expression of just two protein isoforms. Variants 1a and 1b encode a protein of 481 amino acids, while variant Δ1a encodes a shorter 222 amino acid protein (Figure 1). These transcripts have been found in a variety of tissues and have been shown to localise to the cytoplasm of neurons [7]. Recently, the product of this gene was identified as being a homologue of DENN domain proteins (differentially expressed in normal and neoplastic cells), which are GDP/GTP exchange factors (GEF) that activate Rab-GTPases and that are involved in membrane trafficking [45]. 

## 6. Loss-of-Function Mechanism 

The GGGGCC hexanucleotide repeat is located between the two alternative noncoding first exons 1a and 1b. It therefore lies within the promoter region of variant 1b and within intron 1 of variants 1a and Δ1a. In patients studied by DeJesus-Hernandez et al. [7], there was an absence of variant 1b (variant 1 in the original paper) transcribed from the mutant allele, leading to a ~50% reduction in this variant but normal transcription of variants Δ1a and 1a (variants 2 and 3). This finding potentially implicates a loss of gene function as the cause of the disease [2]. Important supporting evidence for this mechanism was found recently by Ciura et al. [46]. They found that morpholino-induced knockdown of the zebrafish homologue of the *C9orf72* gene caused axonal degeneration in motor neurons, leading to behavioural and cellular deficits that affected locomotion without morphological abnormalities. Most importantly, this phenotype was rescued upon overexpression of human *C9orf72* mRNA transcripts. Another important finding that may point towards haploinsufficiency is that large hexanucleotide expansions have been shown to be associated with increased CpG methylation of the *C9orf72 *promoter, which would be expected to lead to downregulation of gene expression [47].

## 7. Gain-of-Toxic-Function Mechanism

Aside from haploinsufficient loss-of-function theories of pathogenesis, several gain-of-function hypotheses have been suggested. One such mechanism may occur through the formation of RNA *foci*, which are known to play an important role in the pathogenesis of several other noncoding repeat expansion disorders, like myotonic dystrophy type 1 [48]. In these disorders, RNA *foci* have been shown to sequester RNA-binding proteins, which lead to dysregulation of alternative mRNA splicing [2, 49–52]. The observation that such RNA *foci* are indeed present in the nuclei of frontal cortex neurons and spinal cord lower motor neurons of c9FTD/ALS patients potentially supports this gain of toxic function mechanism [7].

Another gain-of-function mechanism has been proposed whereby the intronic repeat expansion is translated into dipeptide-repeat proteins through a mechanism of translation not requiring ATG initiation. The translation of the GGGGCC repeat in its three possible reading frames would form poly-(Gly-Ala), poly-(Gly-Pro), and poly-(Gly-Arg). Such dipeptide proteins would be extremely hydrophobic, tending to form intracellular aggregates, and such inclusions have indeed been reported in brain homogenates from c9FTD/ALS patients [53, 54].

## 8. Current Available Therapies for ALS

Riluzole is an inhibitor of glutamate release that acts as a neuroprotective therapy for patients with ALS, and it is currently the only Food and Drug Administration (FDA) approved drug with any proven efficacy to treat this disease. However, the beneficial effects are very modest [55]. Apart from riluzole administration, the management of patients is mostly in terms of symptomatic treatment. Alternative and offlabel treatments are also available and include insulin-like growth factor-1, lithium carbonate [56], minocycline [57], and stem cell therapy [58]. Lithium treatment slowed down progression of the disease in human patients, and in a mouse model, it showed decreased cell death in certain regions and was found to affect multiple targets that may together contribute to the improvement observed [56]. Minocycline has antiapoptotic and anti-inflammatory effects *in vitro* and went to a phase III clinical trial (http://clinicaltrials.gov/ identifier: NCT00047723) but was found to have harmful effects in ALS patients [57]. Antioxidants were also thought to have neuroprotective effects; however, several of them showed no significant effect in clinical trial for this disease [59]. 

## 9. Current Available Therapies for FTD

FTD does not have any FDA approved drugs; however, some offlabel psychiatric drugs are used including antidepressants and some drugs used for Alzheimer's disease [60–62]. For additional indication for FTD, there is an ongoing phase IV clinical trial testing the drug memantine (NCT00545974), which is already approved for use in Alzheimer's disease [60, 61].

## 10. Development of Oligonucleotide-Based Therapy 

Antisense oligonucleotides are short, synthetic, and modified nucleic acids that are able to bind to mRNA or pre-mRNA via base-pairing and to interfere in its function by silencing or through some other form of modulation [63]. ASOs are able to perform their function either by binding to the RNA without inducing RNA degradation or through promoting the degradation of the target RNA via enzymes such as RNase H [64, 65]. RNase H can recognize almost any RNA-DNA heteroduplex, and after this first step, it cleaves the RNA and sets the DNA free. This means that ASOs that can mimic this heteroduplex structure when they bind RNA are also able to recruit RNase H, resulting in cleavage of their target RNA while the ASO is preserved intact. This allows a single ASO to interact with multiple RNAs, enhancing the overall silencing effect [63, 66]. ASOs that act by this mechanism can achieve 80–95% downregulation of protein and mRNA [67]. Other ASOs can also modulate RNA without degradation, for example, through the use of splice modulation to affect the gene product, as is observed for the exon-skipping based therapy currently in development for DMD [68–71]. 

**Figure 2:**
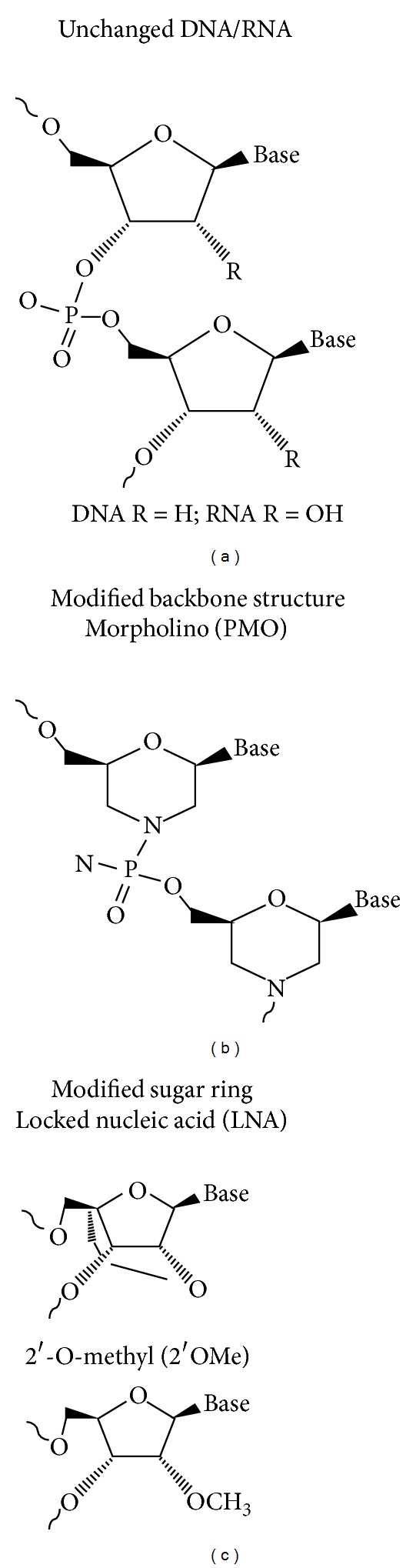
Chemical structures of commonly used oligonucleotides. Nucleic acids can bind to RNA targets that are complementary to their own sequence and trigger target degradation. However, unmodified DNAs/RNAs are subject to endonuclease degradation. Thus, to target RNAs, modifications to this primary structure are needed. Modifications to the nucleic acid backbone can lead to structures that can better interact with the target and are resistant to endonucleases. Modifications in the sugar ring are also possible and give rise to nucleic acids that mimic RNA, have better targeting, and are also resistant to endonuclease action.

To perform their function in an effective manner, ASOs have to reach the mRNA or pre-mRNA in the relevant cellular compartment (e.g., the nucleus) and to successfully hybridize with their target. This requires a molecule with a sufficient half-life and one that is also efficiently taken up by cells. Since unmodified nucleic acids are rapidly degraded by endonucleases and since this may lead to unexpected toxicity or increase in off-target effects if high doses are required, several chemical modifications have been developed to achieve better functioning ASOs [63] (Figure 2).

## 11. Potential ASO Drugs 

**Figure 3:**
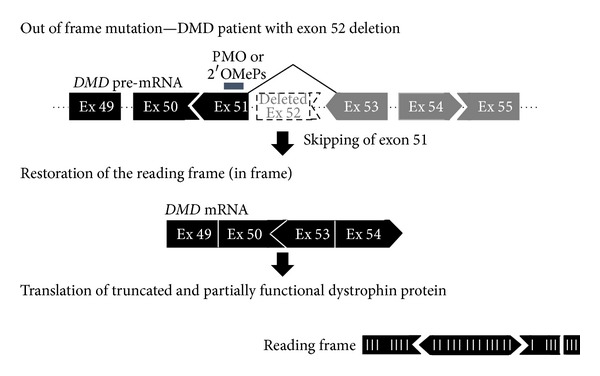
Strategy for exon 51-skipping in Duchenne muscular dystrophy. Exon 51-skipping by appropriate PMO or 2′OMePS, indicated by a blue line, can restore the reading frame of dystrophin in a DMD patient, who lacks exon 52 in the mRNA of the *DMD* gene, leading to out-of-frame products. Dot line and Ex indicate introns and exons, respectively.

Modifications to the phosphate backbone of nucleic acids can give rise to structures more resistant to degradation by nucleases, and this increases the ASO half-life. Particularly important members within this class of modification are the phosphorodiamidate morpholino oligomers (PMOs), which have high resistance to nucleases and are not substrates for RNase H cleavage. PMOs are used for nondegradative purposes such as splice modulation or translational interference [63]. Morpholino antisense oligonucleotides also have some advantages over other types of ASO; they have a higher specificity, and it is suggested that they have fewer off-target effects [72]. PMOs have been successfully used in preclinical research and in clinical trials aimed at exon-skipping in DMD, and currently, a phase IIb exon-skipping clinical trial is ongoing with PMO [68–71] (NCT01396239) (Figure 3). 

Another class of modifications is those made to the sugar ring, which can increase both the RNA-binding affinity and nuclease resistance, leading to enhanced metabolic stability and better pharmacokinetic and toxicological properties [64, 73]. However, such modifications generally lead to loss of RNase H cleavage, and so “gapmer” structures were developed where sugar-modified residues are present either side of a modified backbone ASO. The external sugar-modified residues thus enhance RNA binding, while the internal backbone-modified residues still allow RNase H cleavage [63]. The locked nucleic acid (LNA) is one of the oligonucleotides that has such modifications, and it has great thermal stability, is resistant to exonucleolytic degradation, has good aqueous solubility, and is easy to synthesize [74]. The conformational change observed in the LNA structure mimics RNA and increases target specificity, making this a great therapeutic candidate [75, 76]. In 2005, an LNA-antisense drug to treat chronic lymphocytic leukemia entered phase I/II clinical trials (NCT00285103). Another sugar modification is the 2′OMe modification, which improves thermal stability of the oligonucleotide/RNA hybrid. This alteration, when associated with backbone modifications, can also lead to resistance to endonuclease degradation [73]. The 2′OMe with phosphorothioate modification (2′OMePS) can improve the stability of ASO. Currently, there are clinical trials for exon skipping in DMD using a 2′OMe backbone modified oligonucleotide, including one phase III trial [71, 77] (NCT01254019) (Figure 3). 

## 12. Therapeutic Benefits of Using ASO Drugs

One further attractive benefit to using ASOs as therapy is that they do not need viral or lipid carriers in contrast to other types of RNA-targeting therapy. This reduces the likelihood of an immune response [63]. Most of the ASO applications in development are likely to be achievable through systemic delivery. However, for neurological diseases, there is evidence that direct delivery to the central nervous system may be a better option. In order to reach the brain, systemically delivered ASOs have to cross the blood-brain barrier (BBB). Passage across the BBB is extremely hard for large molecular weight compounds such as ASOs, and to date, no reliably consistent method has been developed that can achieve this. Using currently available ASO chemistries, it is likely that any hope of significant BBB penetration would at the very least require massively increased systemic dosing to a degree that would likely result in toxicity [63, 78, 79]. It has, however, been shown that therapeutic doses of ASOs can be delivered intrathecally in nonhuman primates [80, 81]. This approach, although more invasive than systemic delivery, is routinely used for common clinical indications such as steroid, analgesia, or anaesthesia delivery [82–84] and suggests that this route may be a clinically feasible option. Delivery into the cerebral ventricles is also a possible option, since they contain the cerebrospinal fluid (CSF) that circulates around the brain and spinal cord approximately 3 times a day [81, 85]. Although this approach is invasive and generally requires neurosurgical placement of an indwelling cerebroventricular catheter, it is a route that is on occasion required for clinical drug delivery [86, 87].

## 13. A Perspective on Oligonucleotide-Based Therapy for c9FTD/ALS

ASO therapies can treat gain-of-function disorders by silencing the causative gene, either acting on both alleles for non-essential genes or by selectively acting on the mutant allele in the case of essential genes. For certain loss-of-function diseases, there is also the possibility of using ASOs for splice modulation, which can in certain cases restore the gene function or otherwise compensate for its loss [63]. Since c9FTD/ALS is a disease for which both of these pathogenic mechanisms have been proposed, the use of oligonucleotides for therapy is an attractive option.

In conjunction with this, it is notable that there are several neurodegenerative diseases for which research into ASO therapy is already being performed, including the form of ALS caused by mutations in the *SOD1* gene [81, 88] (NCT01041222). The TDP-43 mutations in familial and sporadic ALS are also a target of knocking down therapies, and recently, the inhibition of a RNA lariat debranching enzyme led to reduced TDP-43 toxicity in yeast and neuronal models [89]. These facts are important because such researches are helping to set the parameters for ASO therapy in ALS and within the central nervous system.

## 14. Therapeutic Potential of Oligonucleotide-Based Therapy for Other Neurodegenerative Diseases

Most importantly, some of the neurodegenerative diseases under investigation are caused by trinucleotide repeat expansions, and evidence of successful ASO therapeutic strategies for this type of disorder is rapidly accumulating. A remarkable example is Huntington's disease, in which an inherited CAG expansion in the *HTT* gene causes progressive chorea, psychiatric disturbance, and generalised cognitive deterioration [63]. Unaffected individuals have up to 35 repeats in the *HTT* gene, while affected patients have from 36 to more than 100 repeats [90]. Trinucleotide repeats are able to form hairpin structures [91], and the different number of repeats in wild type and mutated alleles makes it possible to conduct selective inhibition, since each allele will have a different hairpin structure [92]. In this case, selective silencing is needed, since the *HTT* is an essential gene [93, 94]. In a study performed by Hu et al. [92], selective inhibition of the *HTT* mutant allele with a certain number of repeats was achieved without affecting other genes containing CAG repeats. In the same study, the authors showed that the same approach can also be used for spinocerebellar ataxia type 3, which is another CAG repeat expansion disorder in which the protein affected is ataxin-3 that is involved in deubiquitination and proteasomal protein degradation [95]. These findings show that selective inhibition is feasible for other CAG repeat expansion disorders. Infusion of ASOs into the central nervous system provided long-term reduction in the mutated huntingtin protein, and it was shown that a certain degree of wild type protein suppression can be tolerated [80]. Additional studies have been done to show that repeat expansion disorders like Huntington's disease and spinocerebellar ataxia type 3 can be successfully treated with ASOs [96]. For spinocerebellar ataxia type 3 exon skipping can be performed, since the protein disrupted in this case is essential, and so restoration of its expression is desirable [97]. Another repeat expansion disease that has been shown to be treatable by ASO therapy is myotonic dystrophy type 1, which has a CTG repeat expansion. This disease, most significantly, has the repeat expansion in a noncoding region [98, 99] and is known to form RNA *foci* [100, 101], making it similar to c9FTD/ALS. Applying ASOs that utilize the RNase H mechanism in a transgenic mouse model, gene knockdown and amelioration of pathology were observed [102]. 

**Figure 4:**
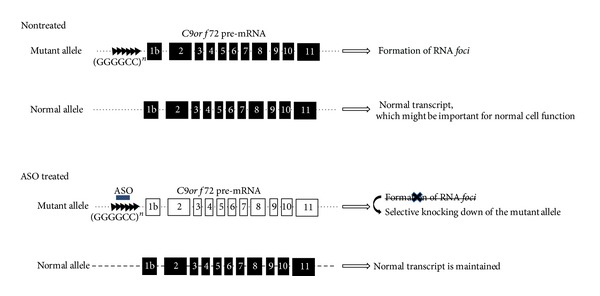
Strategy for ASO-based knocking down of the *C9orf72* gene in a c9FTD/ALS patient. Using antisense oligonucleotides (ASO) targeting the GGGGCC repeat expansion can lead to selective knocking down of the mutant allele which could otherwise cause the formation of RNA *foci*. It also preserves the expression of the normal allele that may have an important function for cell survival. ASO is indicated by a blue line, dot line indicates introns, and squares indicate exons.

The existence of successful studies that were able to ameliorate the effects of repeat expansion diseases shows the feasibility of ASO therapy in such diseases, raising the possibility of developing an ASO therapy for c9FTD/ALS. The possibility of selective suppression of mutant alleles is also a point that has to be considered, since the exact function of the *C9orf72* protein has yet to be studied and since selective inhibition may be important. Our preliminary data suggest that oligonucleotide-based therapy targeting *C9orf72* may be effective in patient-derived fibroblast cells (Figure 4). In addition, the knowledge and expertise obtained from preclinical and clinical studies for the ASO-based treatment of DMD, sporadic ALS, and myotonic dystrophy type 1 could be applicable to c9FTD/ALS in order to develop promising oligonucleotide-based drugs.

## 15. Conclusion

The recent discovery of the hexanucleotide repeat expansion in the *C9orf72* gene as the causative agent of c9FTD/ALS gives rise to the opportunity to develop new therapies directed at this mutation, which is responsible for a large proportion of FTD and/or ALS cases. ASOs are now becoming more widespread in use as gene therapies, and the possibility of chemical modifications that can enhance their properties makes them great candidates for drug development. The recent advances in the development of ASOs therapies for diseases of the central nervous system and in repeat expansion diseases highlight the potential of this approach for targeting the newly discovered c9FTD/ALS mutation. 

## Figures and Tables

**Table 1:** Comparison of pathology, clinical features, and prevalence of distinct FTD and/or ALS causative genes.

	Pathology						Clinical feature	Prevalence	References						
	*TDP-43 *	*P62/ubiquitin *	*Ubiquilin2 *	*FUS *	*SOD1 *	*Tau *			
FTD/ALS *C9orf72 *	+	+	−	−	−	−	ALS—weakness in limbs (50–70%), bulbar involvement, dementia, and psychosis. FTD—behavioural variant, psychiatric symptoms.	60% hereditary ALS-FTD Up to 21% sporadic ALS 7–10% sporadic FTD	[22, 25–28]

FTD/ALS *UBQLN2 *	+	+	+	+	−	−	ALS—upper motor neuron with spasticity, bulbar and pseudobulbar dysfunction, lower motor neuron involvement not prominent, and dementia. FTD—behavioural variant.	5 families	[32, 33]

ALS *FUS *	−	+	−	+	−	−	Lower motor neuron signs with limbs and bulbar distribution, and upper motor neuron signs are common.	4-5% familial ALS 0.5–0.7% sporadic ALS	[24, 29, 30, 34, 35]

ALS *SOD1 *	−	+	−	−	+	−	Lower and upper motor neuron signs, bulbar onset is unusual, and weakness in limbs is asymmetric.	12-13% familial ALS 1–3% sporadic ALS	[24, 36, 37]

ALS *TARDBP *	+	+	−	−	−	−	Weakness in arms before legs, few patients with bulbar involvement, and upper motor neuron involvement with mild or absent spasticity.	3–6% familial ALS 8 patients described sporadic ALS	[24, 38]

FTD tau (*MAPT*)	−	−	−	−	−	+	Behavioural variant, progressive nonfluent aphasia.	Up to 50% of total FTD patients	[39–41]

FTD *FUS *	−	+	−	+	−	−	Behavioural variant.	<1% of total FTD patients	[28, 41]

FTD-TDP (*GRN*)	+	+	−	−	−	−	Behavioural variant, semantic dementia, and progressive nonfluent aphasia.	3–26% of total FTD patients	[15, 36, 41–44]
